# Full-field MRI measurements of in-vivo positional brain shift reveal the significance of intra-cranial geometry and head orientation for stereotactic surgery

**DOI:** 10.1038/s41598-021-97150-5

**Published:** 2021-09-03

**Authors:** Stefano Zappalá, Nicholas J. Bennion, Matthew R. Potts, Jing Wu, Slawomir Kusmia, Derek K. Jones, Sam L. Evans, David Marshall

**Affiliations:** 1 School of Computer Science and Informatics, Cardiff University, Cardiff, UK; 2 Cardiff University Brain Research Imaging Centre (CUBRIC), School of Psychology, Cardiff University, Cardiff, UK; 3 School of Engineering, Cardiff University, Cardiff, UK; 4 Centre for Medical Image Computing, University College London, London, UK; 5 MRI Unit, Epilepsy Society, Chalfont St Peter, UK

**Keywords:** Imaging, Anatomy, Nervous system, Computational models, Computational neuroscience, Databases, Image processing

## Abstract

Positional brain shift (PBS), the sagging of the brain under the effect of gravity, is comparable in magnitude to the margin of error for the success of stereotactic interventions ($\sim$ 1 mm). This non-uniform shift due to slight differences in head orientation can lead to a significant discrepancy between the planned and the actual location of surgical targets. Accurate in-vivo measurements of this complex deformation are critical for the design and validation of an appropriate compensation to integrate into neuronavigational systems. PBS arising from prone-to-supine change of head orientation was measured with magnetic resonance imaging on 11 young adults. The full-field displacement was extracted on a voxel-basis via digital volume correlation and analysed in a standard reference space. Results showed the need for target-specific correction of surgical targets, as a significant displacement ranging from 0.52 to 0.77 mm was measured at surgically relevant structures. Strain analysis further revealed local variability in compressibility: anterior regions showed expansion (both volume and shape change), whereas posterior regions showed small compression, mostly dominated by shape change. Finally, analysis of correlation demonstrated the potential for further patient- and intervention-specific adjustments, as intra-cranial breadth and head tilt correlated with PBS reaching statistical significance.

## Introduction

Given its low stiffness, brain tissue shifts within the skull cavity under the effect of gravity due to changes in head orientation even in normal healthy individuals without any surgical manipulation^1–3^. In this case, displacements of a few millimetres are typically observed, whereas displacements as large as a few centimetres are often observed for pathological causes (e.g. tumour, hydrocephalus) or surgical intervention (e.g. skull or dura opening, cerebro-spinal fluid leakage, device insertion, tissue resection)^4–6^. Commonly referred to as brain shift (BS), displacements are generally comparable if not 2–3 times larger than the current accuracy of image-guided neurosurgical systems (IGNS)^1,2,7^. These systems are routinely used for the planning and navigation of stereotactic procedures such as deep brain stimulation, local drug delivery and stereotactic biopsy^6,8,9^.

Provision of correct neuronal stimulation, drug administration or tissue biopsy requires accurate placement of the probe to within 1–2 mm of the target^10–13^. Despite showing sub-millimetre accuracy^6,9,12^, IGNS usually rely on a global rigid alignment of the pre-operative and the intra-operative scans; this approach implicitly assumes that every structure rotates and translates in an identical fashion and, as such, maintains the same dimensions and shape^3,14,15^. However, the BS caused by any slight differences in head orientation between the pre-operative scanning session and the surgical procedure can cause a non-uniform deformation comparable in magnitude to the margin of error for surgical targeting^1,2,16^. This positional BS (PBS) occurring without any presence of pathology nor surgical manipulation can affect the outcome of a procedure, as the planned targets might differ from their actual location.

Any further improvement of IGNS does not rely on the accuracy of the components used but on correcting surgical targets given the shift arising during a surgical procedure^3,17^. Integrating a reliable compensation of PBS would greatly benefit clinical practise, as the standard for confirming correct surgical targeting can be either too risky (e.g. micro-electrode recording^18^) or too costly (e.g. intra-operative imaging^19,20^). Mathematical models predicting PBS have recently shown promising results^20,21^: given the increasing availability of computational power, such complex models can be run almost in real-time^22–24^. To improve the reliability of such models, datasets of accurate measurements representing such deformation would be invaluable for their development and validation.

Accurate modelling of PBS is enough of a challenge even in normal physiological conditions: displacement is non-uniformly distributed as it is induced by a complex interaction of gravity, anatomical boundaries and fluid pressure; moreover, mechanical response of tissue varies among structures characterised by different histological compositions^1,2,25^. PBS is mostly pronounced in deep, central brain regions (e.g. in the basal ganglia which are the main targets for IGNS-based interventions) and varies even between individual sulci and gyri of the cortex^1,2,16^. The shift resulting from prone-to-supine repositioning has a rotational component in the sagittal plane, with the centre of rotation located around the brainstem region^1,2^. Boundary structures limiting such deformation include: the skull, the falx cerebri, the tentorium cerebelli and the meningeal elements^1,2,26,27^. In addition to acting as a centre of rotation, the brainstem also exerts tension on the brain tissue depending on the angle of the neck, even if this contribution is secondary to the effect of gravity (at neck flexion between $20^\circ$ and $30^\circ$)^28,29^. To the best of the authors’ knowledge, these conclusions were drawn based on observations mostly limited to surfaces^1,2^ or to measurements at specific locations^30,31^.

The aim of this study was to acquire and analyse a dense set of full-field measurements of the in-vivo deformation of brain tissue resulting from a prone-to-supine change in head orientation. Understanding the mechanics in normal physiological conditions (i.e., in the healthy brain) is the first step before modelling the more challenging shift induced by pathology or surgical manipulation. Elastic image registration was used to extract the displacement field between skull-aligned magnetic resonance (MR) scans representing the different states of deformation of the tissue (technique otherwise known as Digital Volume Correlation (DVC)). The study adds to the previous knowledge on PBS^1,2,16,31^ with an analysis based on a set of measurements over the entire brain area; with a quantification of the accuracy of these measurements against a biofidelic synthetic ground truth (reported in the Supplementary Materials); finally, with a normalisation of data from different subjects to a common reference space allowing an inter-subject analysis on a voxel-wise basis. In summary, the novel contributions of the paper are:

- provision of a dataset of accurate volumetric measurements at various regions of interest (ROI) and surgically relevant structures in a standard reference space;
- investigation of the local compressibility of the brain, in particular to further test the hypothesis that the brain is slightly compressible with spatial heterogeneity in compressibility;
- exploration of factors influencing PBS, such as intra-cranial geometry and head orientation.

The reminder of the paper discusses data acquisition and processing as well as normalisation and analysis of deformation. Results prove the comparable magnitude of PBS to the margin of error for the success of IGNS-based interventions. Analysis of deformation casts a light on the heterogeneity of the distribution of the displacement and strain fields, showing the need for target-specific correction of surgical trajectories. Finally, analysis of correlation allows the quantification of the effect on PBS of intra-cranial breadth and head tilt, proving the need for further patient- and intervention-specific adjustments.

## Methods

### Data acquisition

Eleven healthy participants (7 male and 4 female) took part in the study; a narrow and young adult age range (average age: 25.18 years; range: 22–32 years) was chosen to limit the confounding effect of age^2^. The study was approved by the Ethical Committee of the Cardiff University School of Psychology, United Kingdom. All methods were carried out in accordance with the relevant guidelines and regulations. Informed consent was given by all participants before scanning. To ensure that the brain had settled completely to the anterior part of the skull, the protocol included an initial pre-conditioning session, where participants lay face-down for 20 min^1^. Participants were first scanned in the same prone position and then helped to lie in a normal supine position for the following supine scan. Due to scanner preparation, a minimum of 10 min was guaranteed between the swap of participant positioning and the supine acquisition. This was confirmed with an initial pilot study which showed negligible deformation of the brain tissue after around 8 min. Analysis of the time evolution is the future direction of the study, which is underway. For both acquisitions, care was taken to position the head of each participant consistently in the head coil and to locate the latter at the isocentre of the magnetic field, via a laser module built in the system. This was done in order to limit any effect caused by coil location within the magnetic field. The first six participants were scanned in a Siemens 7T MAGNETOM scanner (Siemens Healthcare, Erlangen, Germany); the last five were scanned in a Siemens 3T PRISMA at the same Cardiff University Brain Research Imaging Centre (CUBRIC), Cardiff University. T1-weighted (T1w) MPRAGE sequences^32^ were run for both prone and supine scans. Acquisition parameters are reported in Table 1. Manual shimming was run on the 7T scanner, whereas the vendor’s automatic shimming was used for the 3T data.

**Table 1:** Parameters for the MPRAGE T1w sequences run on 7T and 3T scanners.

	7 T	3 T
TR	2200 ms	2300 ms
TE	2.93 ms	2.88 ms
Flip angle	7$^\circ$	9$^\circ$
FOV	$256\times 318$	$256\times 256$
Slices	242	256
Resolution	$0.8\times 0.75\times 0.75~\hbox {mm}^3$	$1\times 1\times 1~\hbox {mm}^3$
Scan time	4 m 28 s	5 m 32 s

### Data pre-processing

In order to reduce any residual MR distortions, correction with the software *gradunwarp*^33^ was applied (standard for the multi-site Human Connectome Project^34^). Its performance relative to the scanner-default distortion correction was tested on two participants who were scanned in both 7 T and 3 T scanners (see Supplementary Materials). Scans were corrected for low-spatial frequency intensity inhomogeneities with the unified segmentation module of the statistical parametric mapping (SPM)^35^ toolbox^36^. Finally, a semi-automated segmentation process was carried out to extract the skull and brain masks, where the brain extraction tool (BET) command of the FSL software library^37^ was used and the segmentations thus-obtained were amended manually where necessary via Seg3D (Scientific Computing and Imaging Institute (SCI)).

### Digital volume correlation

Prior to elastic registration, images were aligned at the level of the skull in order to define the initial conditions of deformation. The prone scan of each participant was registered to the supine one, which served as the subject-specific reference volume. Registration was limited to the skull to avoid any biases induced by PBS; affine rather than rigid transformation was chosen to reduce any residual distortions^38,39^. The ANTs^40^ affine registration method was used as it showed better performance than two other popular registration software in correcting for some combinations of rotations and translations (see Supplementary Materials).

The warp field resulting from an elastic registration of the skull-aligned prone and supine images depicted the displacement field due to PBS alone^1^. The symmetric image normalisation (SyN)^40^ method was used: it showed better performance than two other state-of-the-art registration packages for neuroimaging in following a biofidelic synthetic deformation field representing PBS (see Supplementary Materials). Parameters were left as default apart from those controlling similarity measure (cross-correlation) and the transformation model (BSpline^41^) which were optimised against the ground truth; the best set gave an error of $0.0342\pm 0.0182$ mm (5th percentile: 0.0109 mm, 95th percentile: 0.0680 mm) in the brain area, one order of magnitude smaller than the expected magnitude of PBS.

### Spatial normalisation

In order to conduct an inter-subject (group) analysis, all supine scans were spatially normalised (that is, elastically registered) to the MNI152 standard space (isotropic resolution 1 $\hbox {mm}^{3}$)^42^ with the same SyN software. Vectors of each displacement field were reoriented (ANTs suite) according to the global rotation matrices representing the specific head orientations of participants relative to the standard space; this guaranteed the correspondence between deformation and head orientation following normalisation^43^. An average displacement field was extracted with the corresponding inter-subject variability that would otherwise be lost when measuring PBS on templates extracted after averaging images between participants^1^.

Furthermore, the normalisation allowed the derivation of the orientation of each participant’s head in the scanner (i.e. direction of gravity) relative to the neutral supine position represented by the MNI152 standard space, as well as the antero-posterior diameter (APD: 176 ± 6 mm) and the maximum cranial breadth (MCB: 137 ± 6 mm)^2^. Any correlation between these factors and PBS was evaluated using the Spearman correlation coefficient^44^.

### Analysis of deformation

Statistics were computed both globally and locally with MATLAB R2020 (Mathworks, Natick, MA, USA). Results presented in throughout the paper are either reported in Cartesian or in spherical coordinates. For the former, a RAS (right-anterior-superior) convention was used. For the latter, the azimuth angle represented orientation of vectors on an axial plane relative to the positive axis of the left–right (L–R) direction; elevation angles represented orientation on a sagittal plane relative to the positive axis of the posterior–anterior (P–A) direction.

A ROI-wise analysis was performed after normalisation to infer the anatomical variability in PBS. Atlases used include: Harvard Oxford (HO)^45^, the Atlasing of the Basal Ganglia (ATAG)^46^ and the International Consortium for Brain Mapping (ICBM)^47^ atlases. These included deep white matter structures as well as ventricles and basal ganglia, which are relevant surgical targets for IGNS-based interventions^48–50^.

Finally, the Green–Lagrange strain tensor^51^ was evaluated at each voxel in the brain in order to interpret the deformation in a differential manner, that is, discarding any rigid body displacement. The strain tensor, $\mathbf{E}$, was extracted as:

1 $$\begin{aligned} \mathbf{E}= & {} \frac{1}{2} \left( \mathbf{F} ^{T}{} \mathbf{F} -I \right) , \end{aligned}$$

2 $$\begin{aligned} \mathbf{F}= & {} \dfrac{\partial \mathbf{u} (\mathbf{x} )}{\partial \mathbf{x} } + I, \end{aligned}$$

where $\mathbf{x}$ is the voxel position, $\mathbf{u} (\mathbf{x} )$ the deformation field, *I* the identity matrix and $\mathbf{F}$ the deformation gradient.

To further investigate the role of the tissue meso-architecture, strain tensors were partitioned into a hydrostatic component representing volume change (at small strains):

3 $$\begin{aligned} E_{hyd}=\frac{E_{xx}+E_{yy}+E_{zz}}{3}, \end{aligned}$$

where $E_{xx}$, $E_{yy}$, $E_{zz}$ are the three diagonal elements of the strain tensor; and a deviatoric component representing shape change:

4 $$\begin{aligned} \mathbf{E} _{dev}=\mathbf{E} -E_{hyd}. \end{aligned}$$

## Results

### Subject positioning

**Figure 1:**
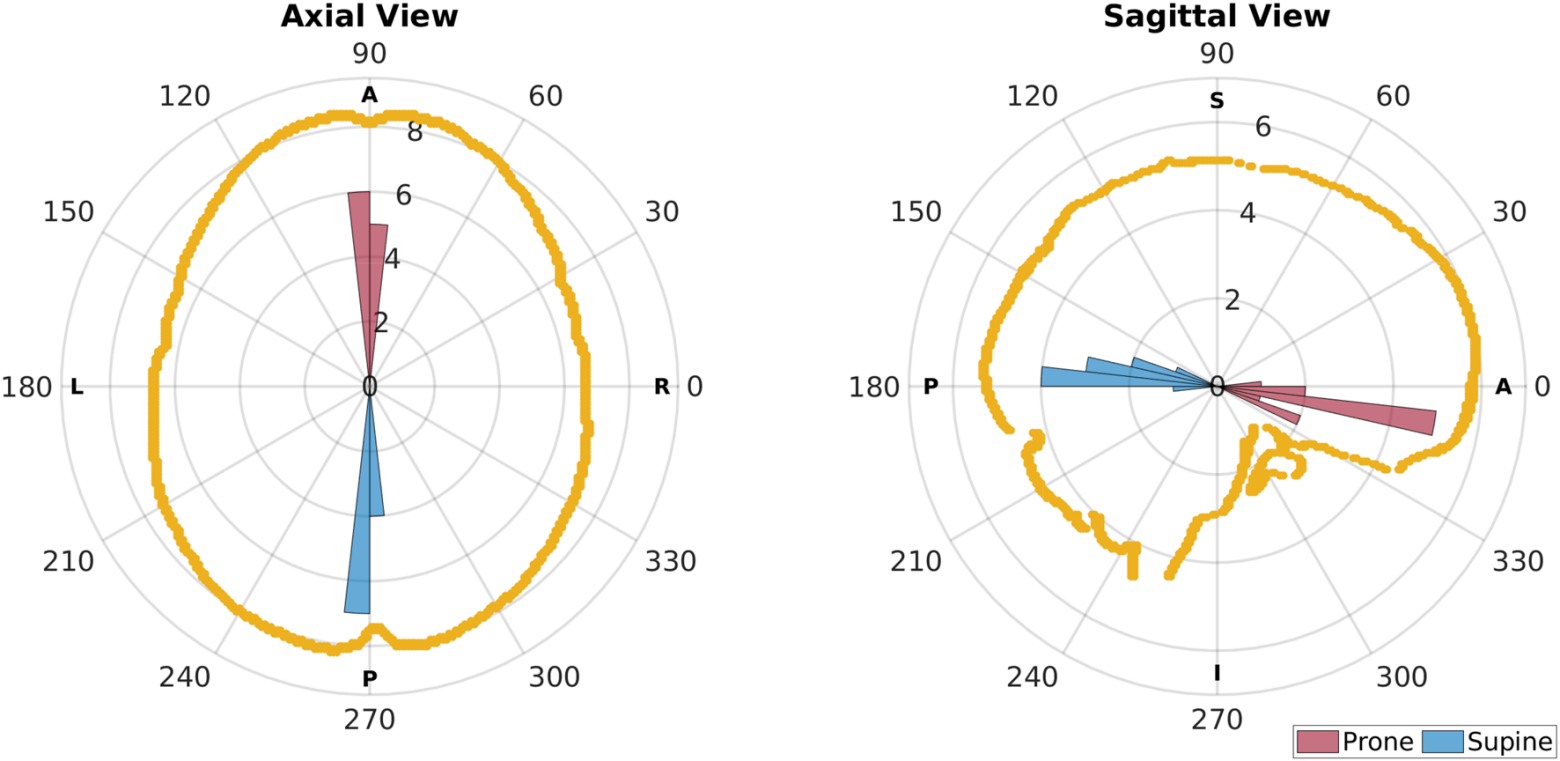
Polar histograms of the direction of gravity (i.e., head orientation of participants) during scanning. On the left, greater values of azimuth angle represent head of the participant turned right during scanning. On the right, higher values of elevation angle represent head of the participant tilted downwards during scanning. The shape of the skull from the MNI atlas is overlapped as reference for the neutral head orientation.

Figure 1 shows head orientation among subjects as direction of gravity during scanning. Average ± standard deviation of azimuth and elevation angles were, respectively, $89.91\pm 2.99^\circ$ and $-9.86\pm 8.90^\circ$ for prone, and $-91.01\pm 2.03^\circ$ and $171.72\pm 6.22^\circ$ for supine, showing an average neutral pan but slight upward tilt of the head in both prone and supine scans.

### Analysis of deformation

#### Overall displacement

**Figure 2:**
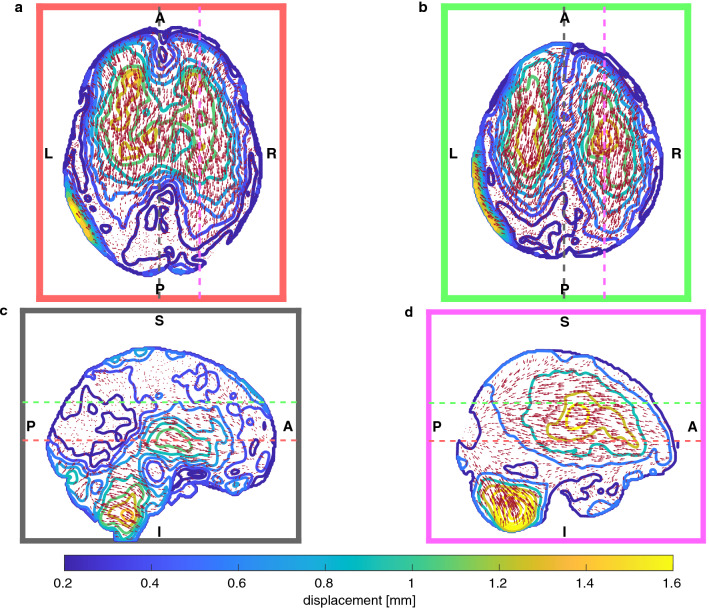
Vector plots of PBS for two axial (*a* and *b*) and two sagittal (*c*, *d*) slices. Length of vectors have been scaled for visualisation purposes: their magnitude is represented by the underlying contour plots. As reference, dashed coloured lines represent the position of the other slices. In particular, slice *a* was positioned at the level of the anterior and posterior horns of the lateral ventricles, whereas slice *c* was positioned at the level of the falx cerebri.

The most significant component of displacement was P–A as reported in Table 2. With respect to the average, the inferior–superior (I–S) component showed greater variability among subjects. A consistent shift towards left can be seen in the L–R component. Figure 2 shows PBS as a displacement field. An overall translational component towards the posterior part of the skull can be seen, with significant local variability. Displacement was greater in deeper (compared to more superficial) regions and in particular further away from anatomical boundaries such as the falx cerebri (midline), the tentorium cerebelli (just below the cerebrum) and the meninges (periphery). Whilst inward displacement can be seen in frontal regions, movement was negligible more posteriorly. The previously observed shift to the left can be seen here in the axial distributions of Fig. 2; this lateral displacement correlated weakly with brain volume ($p=0.07, r=-0.58$), showing a greater leftward deformation with bigger brain volumes.

**Table 2:** Average and standard deviation displacement values in the brain area with the corresponding inter-subject variability. Global statistics are extracted for the three components separately and for the magnitude of displacement.

	Mean ± Standard deviation (mm)	Inter-subject variability (mm)
Left–right	− 0.09 ± 0.23	0.19
Posterior–anterior	− 0.2 ± 0.36	0.26
Inferior–superior	0.10 ± 0.33	0.3
Magnitude	0.57 ± 0.34	0.41

#### ROI analysis

**Figure 3:**
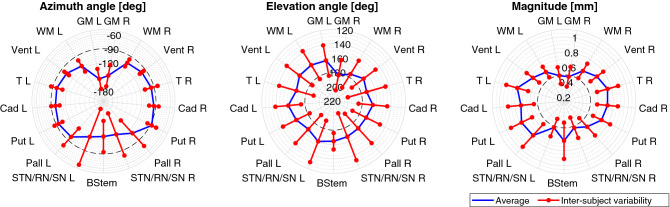
Polar diagrams showing azimuth (left) and elevation (centre) angles as well as magnitude (right) of PBS averaged over some ROI: left (L) and right (R) gray matter (GM), white matter (WM), ventricles (Vent), thalamus (T), caudate (Cad), putamen (Put), pallidus (Pall), subthalamic nucleus (STN), red nucleus (RN), substantia nigra (SN) and brain-stem (BStem). Whiskers represent inter-subject variability. Decreasing values of azimuth angle in the $[-90,-180]^\circ$ range represent vectors progressively oriented towards left, whereas increasing values of elevation angle in the $[90,270]^\circ$ range represent vectors progressively oriented downwards. STN, RN and SN were combined together due to the small number of voxels represented by these structures.

Figure 3 shows deformation within significant ROI. Values of azimuth angle (left of Fig. 3) indicate an overall displacement from anterior to posterior, as well as a predominant leftward component of deformation at peripheral (GM) and inferior (STN/RN/SN and BStem) regions. Elevation angle (centre of Fig. 3) shows an overall upward displacement which was bigger in the left than in the right hemisphere. Magnitude (right of Fig. 3) was greater in deep regions (e.g. basal ganglia) and lesser towards the skull and slightly bigger in the left than in the right hemisphere. Inter-subject variability between ROI showed slightly bigger values at deep structures than at the periphery, with an average of 0.15 mm at surgically relevant ROI.

#### Strain distribution

**Figure 4:**
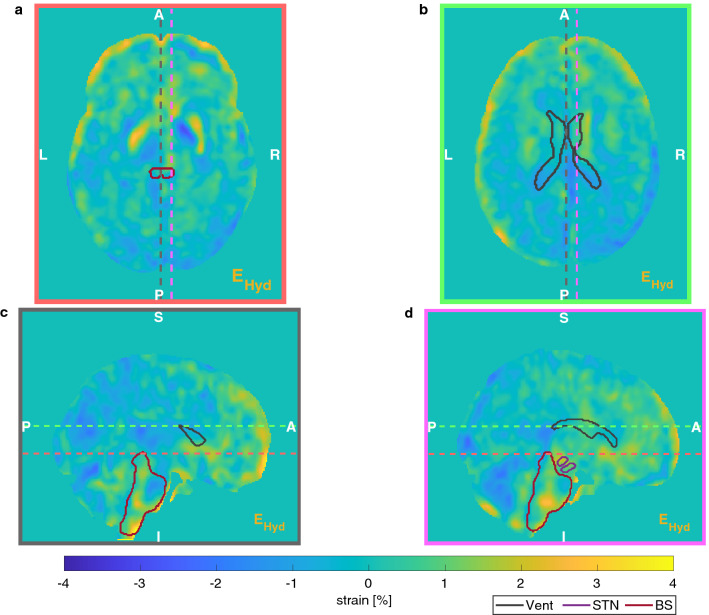
Distribution of the hydrostatic component at two axial (*a* and *b*) and two sagittal (*c* and *d*) slices. As reference, three ROI (Vent, STN, BStem) are delineated. Dashed coloured lines represent the position of the other slices. In particular, slice *a* was positioned at the level of the anterior and posterior horns of the lateral ventricles, whereas slice *c* was positioned at the level of the falx cerebri.

**Figure 5:**
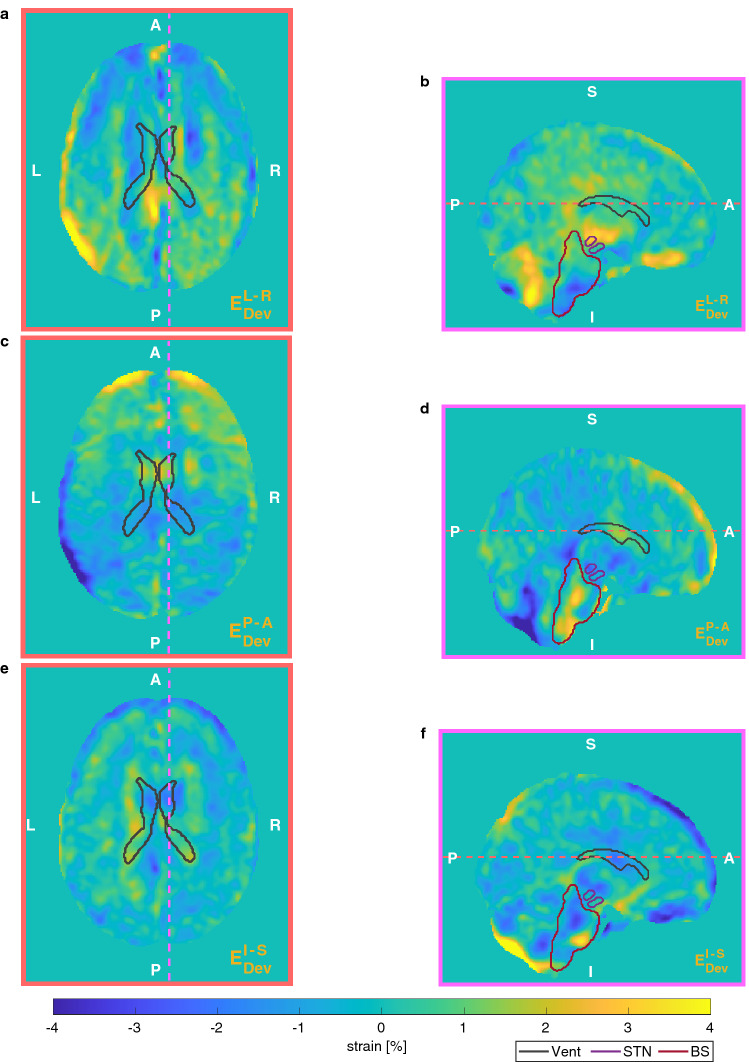
Distribution of the main deviatoric components of the Green–Lagrange strain. First row shows axial (*a*) and sagittal (*b*) slices of the L-R component; second row shows the P–A component (*c* and *d*); third the I–S component (*e* and *f*). As reference, three ROI (Vent, STN, BStem) are delineated. Dashed coloured lines represent the position of the other slices. Axial slices were positioned at the level of the anterior and posterior horns of the lateral ventricles, whereas sagittal slices were positioned 1 cm to the right of the falx cerebri.

**Figure 6:**
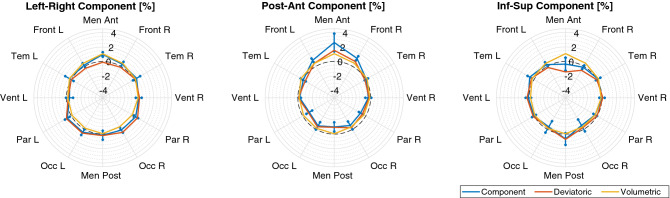
Diagonal components of strain averaged over some ROI: left (L) and right (R) anterior and posterior meninges (Men Ant, Men Post), frontal lobe (Front), temporal lobe (Temp), ventricles (Vent), parietal lobe (Par), occipital lobe (Occ). Blue lines represent the overall diagonal component (whiskers representing inter-subject variability), whereas the orange and yellow lines its deviatoric and volumetric components.

Average and standard deviation values of strain are reported in Table 3, alongside the corresponding inter-subject variability. Figures 4 and 5 show the distribution of the hydrostatic and the deviatoric strains, respectively. Strain maps show elongation in frontal regions and confirm the negligible deformation in posterior regions as previously noticed. Local variability of deformation can be seen, as well as some structures (such as ventricles) and anatomical boundaries (such as the falx cerebri and the tentorium cerebelli). The polar plots in Fig. 6 show the diagonal components of the strain tensor for different lobes also decomposed in its hydrostatic and deviatoric components. Deformation along P–A direction occurred as both volume preserving ($0.52\pm 1.02$ %) and volume change ($0.44\pm 0.64$ %) expansion in the frontal lobe. However, deformation occurred predominantly as shape change in more posterior regions ($-0.48\pm 1.14$ %), with a small volumetric compression ($-0.25\pm 0.76$ %).

**Table 3:** Average and standard deviation values of strain in the brain area with the corresponding inter-subject variability along the three main directions.

	Mean ± Standard deviation (%)	Inter-subject variability (%)
L–R	0.18 ± 1.34	0.09
P–A	− 0.04 ± 1.38	0.08
I–S	0.03 ± 1.32	0.09

### Influence of geometry

**Figure 7:**
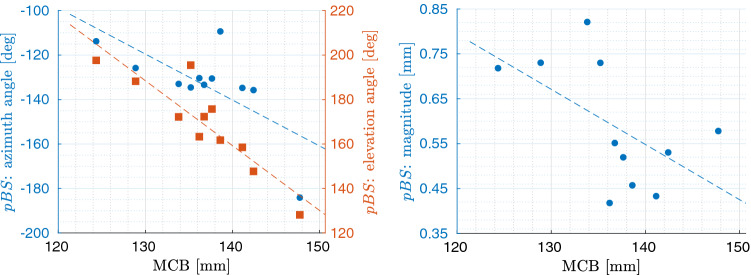
Scatter plots of the correlation between PBS and MCB. Azimuth (blue dots) and elevation (orange squares) angles are reported on the left against values of MCB, whereas against magnitude of PBS on the right. As reference, linear fit is superimposed to the data.

The APD did not reach statistical significance in the correlation with PBS. In supine positioning, however, MCB strongly correlated with azimuth ($p=0.04, r=-0.63$, Fig. 7) and elevation angles ($p<0.01, r=-0.87$, Fig. 7) of PBS, and weakly with magnitude ($p=0.08, r=-0.55$, Fig. 7). Linear fit showed that an increase of 10 mm of MCB led to a displacement $20.66^\circ$ more to the left, a displacement $29.17^\circ$ more downwards and finally a decrease of 0.12 mm in its magnitude.

### Influence of head orientation

**Figure 8:**
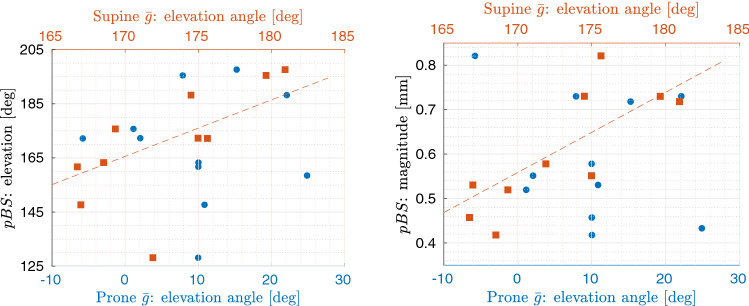
Scatter plots of the correlation between PBS and gravity (${\bar{g}}$). Elevation angle of gravity is here reported against elevation angle (left) and against magnitude of PBS (right) for both prone (blue dots) and supine (orange squares) positioning. As reference, linear fit is superimposed to the data in the case of the statistical correlation between elevation angle of ${\bar{g}}$ in supine and both elevation angle (on the left) and magnitude (on the right) of PBS.

Head orientation in prone position did not reach statistical significance in the correlation with PBS. In supine positioning, however, a statistically significant correlation ($p=0.01, r=0.7364$, Fig. 8) was found between elevation angle of gravity in supine and that of PBS: head tilt $10^\circ$ more downwards in supine induced a shift $10.86^\circ$ more downwards. Moreover, elevation angle of gravity strongly correlated with the magnitude of PBS ($p<0.01, r=0.80$, Fig. 8): head tilt $10^\circ$ more downwards induced a decrease in the shift by 0.18 mm.

## Discussion

The present study successfully captured PBS as three-dimensional deformation over the entire brain volume, without limiting the analysis to any surface (such as the ventricular or the cortical surfaces^1,2^). Differently from previous studies investigating the phenomenon in analogous conditions, accuracy of the measurements was evaluated, giving an error in following a biofidelic ground truth of $0.0342 \pm 0.0229$ mm in the brain area. Analysis showed local variability in both displacement and compressibility of the tissue, demonstrating the complexity of PBS as interaction of gravity, anatomical boundaries and mechanical response of the tissue. Finally, the study revealed a strong correlation between the shift and both head orientation and the geometry of the intra-cranial cavity, giving a measure of their effect on PBS.

Values measured in the present study might be negligible relative to the typical shift seen during more invasive procedures such as craniotomy and tumour resection^14,25,52^. The obtained magnitude, however, was comparable to the error allowed for the correct targeting in IGNS-based interventions^6,9,11,12^: deformation of some surgically relevant structures ranged from 0.52 mm at the STN/RN/SN complex to 0.77 mm at the T. Values were in accordance with previous studies investigating the phenomenon in analogous conditions. Among these, Hill et al.^16^ compared scans for two patients before surgery finding a deformation smaller than the resolution of their scans (1 mm). Schnaudigel et al.^1^ reported brain deformation between 0.6 and 1.3 mm. Monea et al.^2^ reported a $95\%$ confidence interval of inwards shift between 1.08 and 0.47 mm at the brain surface and between 0.72 (inwards) to 0.83 mm (outwards) at the ventricular surface. Rice et al.^30^ reported a value of PBS of 1 mm from measuring the change in thickness in occipital cerebro-spinal fluid. Recently, Yokoyama et al.^31^ reported a downward and posterior displacement of the pituitary body of $0.68\pm 0.27$ mm and $0.76\pm 0.24$ mm, respectively, and a shortening of the pituitary gland by $1.23\pm 0.71$ mm from a sitting-to-supine change of positioning.

Deformation happened predominantly along the P–A axis following the direction of gravity, with a lateral component consistent among all subjects. Greater deformation could be seen further away from anatomical boundaries, and in particular in deeper structures, such as T, BG and BStem, confirming the influence of both gravity and anatomical constraints reported in the literature. The joint effect of the curved shape of the skull and the anchoring effect of the BStem most likely induced the anticlock-wise rotation around the L–R axis^1,2,53^; simultaneously, the tethering effect created by the meningeal and vascular elements might have contributed to the smaller deformation near these cortical areas^26,27,54^. The falx cerebri most likely limited any shift along its surface, inhibiting any deformation along the L–R direction (in particular at the level of the WM)^1,2^; Finally, the tentorium cerebelli reduced the I–S deformation of the lowermost part of cerebrum. Results supported the pattern of deformation reported by Schnaudigel et al.^1^; but contrasted Monea et al.^2^, who found a bigger shift of the cortex relative to the ventricles. The observed difference in elevation angle and magnitude of displacement between hemispheres can be related to the reported lateral asymmetry of the intra-cranial cavity^55,56^, as the head pan of subjects during scanning was consistent ($89.91\pm 2.99^\circ$) and did not show any statistical significance in the correlation with PBS.

Strain analysis showed deformation as prevalent shape change rather than actual volumetric compression/extension. Frontal regions showed both stretch and expansion of tissue (consistent with the softer response in tension^57,58^); on the other hand, posterior regions showed a prevalent deviatoric contraction along P–A (accommodated by an elongation along L–R and I–S directions) with a volumetric compression that was half the same component in frontal regions (consistent with the nearly incompressible nature of the brain tissue^59,60^). The compression found in this study can be related to interstitial fluid redistribution and intracellular interactions as water escaping from ex-vivo specimens was reported during pre-conditioning before compressive testing^60^. Values were in accordance with the decrease in volume by $5.07\pm 3.24$ % reported by Yokoyama et al.^31^ at the lateral ventricles from a sitting-to-supine change of positioning. Whilst direct comparison is limited, Libertiaux et al.^61^ reported a standard deviation of up to $5\%$ in the volume ratio of ex-vivo specimens opposing to a natural compressive strain of up to 0.22 at rates between 1.2 and 120 mm/min; Franceschini et al.^62^ showed small deformation of $2.8\pm 1.26 \%$ of specimens under load of 3 and 6 N until displacement died out under physiological saline and free drainage. Conclusions on the mechanical response of the brain tissue are limited by the numerous factors influencing the full-field displacement measured in this study. However, brain tissue showed local variability in volumetric compression (up to 2%) in physiological conditions: these values help in assessing the degree of incompressibility^59,61,63^ to assume when modelling the brain response in light of the accuracy required for the specific application.

The study reaffirmed the lateral component of deformation ($-0.09\pm 0.23$ mm) in prone-supine change of positioning as firstly reported by Schnaudigel et al.^1^. Despite being comparable to the margin of error of the measurements taken, this component was consistent in all subjects and stronger in deep and posterior regions (such as BG)^1^. As head orientation in an axial plane was neutral during scanning, gravity alone could not be the only cause. First, MR distortions are reported to induce a spurious deformation along the L–R axis, which was measured on two phantoms giving absolute differences of $0.4 \pm 0.2$ mm on 7 T^64^ and $1.3 \pm 0.26$ mm on a 3 T scanner^65^ after correction. Second, asymmetry of the hemispheres is a well known characteristic of the human brain (Yakovlevian torque) which presents an anti-clockwise rotation around the P–A axis caused by a bigger right frontal and left occipital lobes relative to their controlateral^55,66,67^. Therefore, the leftward component of deformation seen in the present study could have been a joint effect of both residual distortion after correction and a clockwise deformation when moving to a supine position as a result of an even stronger twist effect when in prone positioning. Moreover, bigger brain volumes showed stronger leftwards deformation, the tissue being less constrained by anatomical boundaries. The results of the study further demonstrate the complexity of the phenomenon, as even an off-axis deformation can be critical to the overall accuracy of its prediction.

The inter-subject variability extracted in this study (average at surgically relevant ROI: 0.15 mm) represents the effect on PBS of further subject- and intervention-specific characteristics that needs to be addressed when modelling such phenomenon. Among these, intra-cranial geometry and head orientation revealed a strong correlation with PBS. Regarding the former, bigger cranial breadths diminished the constraining effect of anatomical boundaries on the brain tissue, giving bigger leftward and upward component of displacement. Monea et al.^2^ also reported a statistically significant correlation of both MCB; however, statistical significance was reached only for lateral PBS and not for prone-to-supine change of positioning. Regarding head orientation, bigger shift was captured in neutral positions relative to more downwards tilted positions, as the curved shape of the skull might have limited the deformation of the brain tissue in more angled head orientations. No significant correlation was found for the head pan, due to the limited range of rotations acquired on an axial plane. In an experimental scenario, these factors need to be addressed in order to increase the accuracy of model-based predictions, conditional to the margin of error for the specific application. For the case of IGNS, for instance, results of the study demonstrate the need for both subject- and intervention-specific correction of surgical trajectories as anatomical differences and slight changes in head tilt on a surgical table can affect the successful targeting of the correct structure.

A significant effort was made to understand and limit the potential inaccuracies related to the measurements, leading to the following main sources of error: residual MR distortions, improper initial skull alignment and inaccuracy of the elastic registration. Phantom and clinical studies on MR distortions report a spurious warp of around 1 mm^64,65,68,69^ even after correction. This warp depends not only on the scanner (static magnetic field inhomogeneities) or the gradient coil (gradient nonlinearities, eddy currents), but also on the scanned object (chemical shift, susceptibility differences)^39,64,65,69^. Distribution of these properties over the brain is non-uniform, with larger distortions in inferior and frontal areas, close to air-filled cavities^38,39,64^. It is not stated whether distortions were accounted for in similar studies measuring PBS, as 1.5 T^1^ or 3 T^2^ scanners were used without any additional CT image. In the present study, *gradunwarp* showed better performance relative to the uncorrected scans and to the scanner-default distortion correction methods when comparing 7–3 T images of two subjects (see Supplementary Materials). Given the complexity of the phenomenon and the lack of a proper correction in the scanner^68^, any further attempt to model distortions was considered out of scope and therefore a limitation of the study. Alongside distortions, any residual differences in the alignment of the skulls between the prone and supine scans of the same subject were captured by the elastic registration as an additional spurious component of deformation. Therefore, accuracy of this first step was evaluated on synthetic images (see Supplementary Materials). Finally, with the aim of minimising the error when approximating the true deformation field, the transformation models of three state of the art elastic registration methods were optimised against a biofidelic ground truth and their performance compared (see Supplementary Materials).

Finally, the present paper is limited by the small sample size, consisting solely of healthy and young individuals. This choice was made for practical reasons, to make an initial evaluation and to limit the confounding effects of age^2^. As such, results presented here cannot be easily generalised to represent the average population of patients necessitating IGNS-based interventions. Widening the age range of the population sample or including patients in the analysis will be of more clinical interest and is going to be a future investigation.

## Conclusion

In the present study, the physiological deformation of the brain tissue under the effect of gravity due to prone-to-supine change of positioning was captured and investigated for a sample of 11 young adults. For the first time, an average volumetric vector field with the corresponding inter-subject variability was extracted, allowing tissue displacement within surgically relevant ROI to be characterised.

Results show that even in the healthy brain without any surgical manipulation, the magnitude of PBS can be comparable to the margin of error for the success of stereotactic intervention, with a significant displacement ranging from 0.52 to 0.77 mm at surgically relevant structures. Although likely confounded by MR distortions, strain analysis confirmed both the reported softer response of the brain tissue in tension ($0.44\pm 0.64$ % volume preserving and $0.52\pm 1.02$ % volume change) and its nearly incompressibility ($-0.48\pm 1.14$ % volume preserving, $-0.25\pm 0.76$ % volume change). Analysis of correlation revealed that cranial breadths 10 mm bigger induced a shift $20.66^\circ$ more to the left and $29.17^\circ$ more downwards, respectively, as well as a decrease in magnitude by 0.12 mm (the latter, with weak correlation). On the other hand, head tilt $10^\circ$ more downwards induced a shift 0.18 mm smaller and $20.86^\circ$ more downwards.

The present study gave a measure of the influence of tissue compressibility, intra-cranial geometry and head orientation on PBS: these factors need to be addressed when modelling such phenomenon depending on the margin of error allowed for the specific application. For the case of deep brain stimulation, drug delivery and tissue biopsy, the stringent 1 mm margin of error necessitates patient- and intervention-specific correction of surgical trajectories to integrate into IGNS before further improving the accuracy of other components. The full vector field extracted in the study is of critical value for the initial validation in simple physiological conditions of any appropriate compensation to integrate into IGNS, before moving to the more complex deformation induced by surgical manipulation.

## Supplementary information

Supplementary Information.

## Data Availability

The dataset generated during and/or analysed during the current study are available in the following OSF repository 10.17605/OSF.IO/GDB29.
