# Supplementary material for: Full-field MRI measurements of in-vivo positional brain shift reveal the significance of intra-cranial geometry and head orientation for stereotactic surgery
Source: Sci Rep. 2021 Sep 3;11:17684. doi: 10.1038/s41598-021-97150-5 (PMC8417262; doi:10.1038/s41598-021-97150-5)
Supplement: Supplementary file 1 — Supplementary Information. [file 41598_2021_97150_MOESM1_ESM.pdf]

Full-field MRI measurements of in-vivo positional brain shift reveal the significance of intra-cranial geometry and head orientation for stereotactic surgery: supplementary materials

Stefano Zappalá[1,2,*], Nicholas J. Bennion[3], Matthew R. Potts[3], Jing Wu[1], Slawomir Kusmia[2,4,5], Derek K. Jones[2], Sam L. Evans[3], and David Marshall[1]

[1]School of Computer Science and Informatics, Cardiff University, Cardiff, UK
[2]Cardiff University Brain Research Imaging Centre (CUBRIC), School of Psychology, Cardiff University, Cardiff, UK
[3]School of Engineering, Cardiff University, Cardiff, UK
[4]Centre for Medical Image Computing, University College London, London, UK
[5]MRI Unit, Epilepsy Society, Chalfont St Peter, UK
[*]email: zappalas@cardiff.ac.uk

The following supplementary materials report the effort that was made to quantify the main sources of error affecting the measurements taken and therefore the conclusions drawn. To the best of the authors' knowledge, this is the first study reporting the accuracy of the measurements taken among those investigating the same phenomenon in homologous conditions [1,2]. First, the relative performance of the distortion correction method used in the study was evaluated against the scanner default software at both 3T and 7T. Second, the accuracy of the initial skull alignment was inferred against some synthetic rotations and translations. Last, three elastic registration methods were optimised against a synthetic deformation field representing positional brain shift (PBS) to extract the best performing one and the corresponding error.

MR Distortion Correction

The performance of the distortion correction *gradunwarp*[3] used in this study was compared to the scanner-default method on two subjects who were scanned both with the 7T and 3T scanners. The performance was tested in relative terms, as the lack of a distortion-free ground truth made it impossible to quantify the absolute warp caused by MR distortions. Prone images acquired with different scanners were warped together via elastic registration (symmetric image normalisation (SyN)[4]), prior to any correction as well as after correction with the scanner default software and with *gradunwarp*. The same was done for the supine scans. Differences between images were assessed in terms of magnitude of the warp field as output of the elastic registration.

Results (Table 1) showed a better correspondence between scans when using the software *gradunwarp*. In prone position, the warp field representing differences between scans was 33% smaller after using *gradunwarp*, and 30% smaller after using the scanner-default software; in supine position, these values were 7% and 6%, respectively. A considerable residual warp has to be acknowledged, which could have been a combination of residual distortions, registration error and the likely presence of PBS due to slight differences in head orientation between sessions. Nevertheless, results are in line with previous studies, for instance: Watanabe et al.[5] reported an improvement in the root mean squared error of 15% after using the default distortion correction with a 3T scanner. Karger et al.[6] reported an increase of 54% in mean deviation with a 3T scanner and 12% with a 1.5T scanner. Tavares et al.[7] reported an improvement of 43% with a 1.5T scanner. Finally, Neumann et al.[8] found an increased accuracy of 6% with 3T and up to 55% with 1.5T scanners after applying the distortion correction.

Skull Alignment

The error given by the skull alignment step (i.e. setting the initial conditions of deformation) was determined against few synthetic transformations. The three affine registration methods from the following software libraries were compared: FSL[9], ANTs[4] and *elastix*[10]. Typical rotations and translations that can be found in the study were represented. A maximum rotation of 30° was applied around the left-right axis (tilt), 5° around the posterior-anterior one (roll) and 10° around the interior-superior axis (pan). Translation was kept to a maximum of 5 mm, given the initial alignment of the centre of images implemented in the registration algorithms. Few compositions of these were also tried. Transformations (Table 2) were applied to the supine scans of 8 subjects prior to the skull-based registration.

Correction	Prone	Supine
None	1.88 ± 0.34 mm	1.40 ± 0.36 mm
Scanner default	1.32 ± 0.11 mm	1.31 ± 0.33 mm
gradunwarp	1.25 ± 0.10 mm	1.30 ± 0.20 mm

Table 1. Differences between scans acquired with the 7T and 3T scanners for both prone and supine positioning prior to correction and after correction with the scanner default software and with *gradunwarp*. Differences are represented in terms of average and standard deviation magnitude of the warp field in the brain area.

	**L-R angle**	**P-A angle**	**I-S angle**	**L-R translation**	**P-A translation**	**I-S translation**
T_1	30°	0°	0°	5 mm	0 mm	0 mm
T_2	0°	5°	0°	0 mm	5 mm	0 mm
T_3	0°	0°	10°	0 mm	0 mm	5 mm
T_4	-30°	0°	0°	-5 mm	0 mm	0 mm
T_5	0°	-5°	0°	0 mm	-5 mm	0 mm
T_6	0°	0°	-10°	0 mm	0 mm	5 mm
T_7	15°	-2.5°	-5°	2 mm	-2 mm	2 mm
T_8	15°	-2.5°	5°	2 mm	2 mm	-2 mm
T_9	-15°	-2.5°	5°	-2 mm	2 mm	2 mm
T_{10}	-15°	2.5°	-5°	-2 mm	2 mm	-2 mm

Table 2. Rotation and translation values tested for the validation of the skull alignment.

The accuracy of this initial step was evaluated by calculating the Dice coefficient, given by[11]:

$$\frac{|skull_{original} \cap skull_{registered}|}{|skull_{original}| + |skull_{registered}|}, \quad (1)$$

where $skull_{original}$ represents the original skull segmentation and $skull_{registered}$ the skull segmentation after registration of the synthetic images.

Fig. 1 shows the dice coefficient averaged among the subjects for each of the registration methods tested. ANTs performed best given the outliers showed by *elastix* (reported in the zoom out box in the bottom-right part of the figure), and was therefore used throughout the study.

Elastic Registration

Three state of the art registration algorithms for neuroimaging were optimised and then compared in order to gauge their accuracy in measuring a synthetic displacement field replicating PBS. The extensive comparisons by Klein et al.[12], Ou et al.[13] and Murphy et al.[14] put SyN[15], *elastix*[10] and DRAMMS[16] at the top for best performance. Registration methods based on mechanical models were discarded not to impose any a priori constraints on the deformation[17, 18]. A realistic displacement field was generated through a biofidelic finite element simulation of PBS (Fig. 2)[19]. This displacement field was characterised by a magnitude of 0.60 ± 0.26 mm, azimuth angle of $-89.70 \pm 11.98°$ and elevation angle of $1.27 \pm 11.46°$. The warp field was applied to the supine scans of 8 participants and these registered back to the original using each of the selected methods. Parameters controlling for the transformation model and the similarity measure were optimised, leaving the others as default in order to reduce the number of combinations to test (Table 3). Regarding the call to *elastix*, a similar parameter file as in Staring et al.[20] was used. Computations were run on a cluster at Cardiff University Brain Research Imaging Centre (CUBRIC). First, the root mean square error (RMSE) between the estimated warp and the ground truth was extracted in the brain area, as well as at some regions of interest (ROI). Sensitivity analysis (MATLAB R2020, Mathworks, Natick, MA) was then used to assess the influence of parameters based on partial correlation with Spearman ranks. Results are reported in Table 4, showing that the most influencing parameters for all methods were the ones controlling for the spacing of control points of the transformation models. Furthermore, a score-based system was implemented, where a parameter's performance was calculated, for every value, as sum of the rank of each optimisation run in terms of accuracy. This was done to avoid any averaging among subjects or ROI (Fig. 4, 8, 6). The optimal set was therefore chosen as the one achieving the smallest RMSE, alongside guaranteeing that the parameter with the greatest sensitivity on the error had the lowest rank score (Fig. 3, 7, 5).

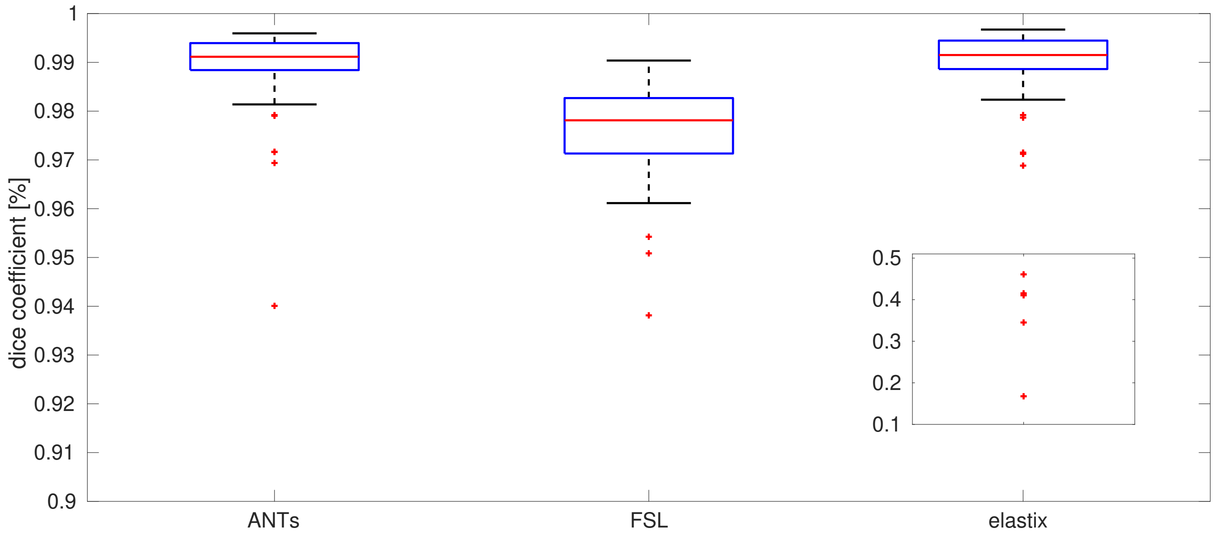

Figure 1. Boxplot of the dice coefficient representing the alignment of skulls obtained for the three registration algorithms tested. In the bottom-right corner a zoom out box highlights few outliers for *elastix*.

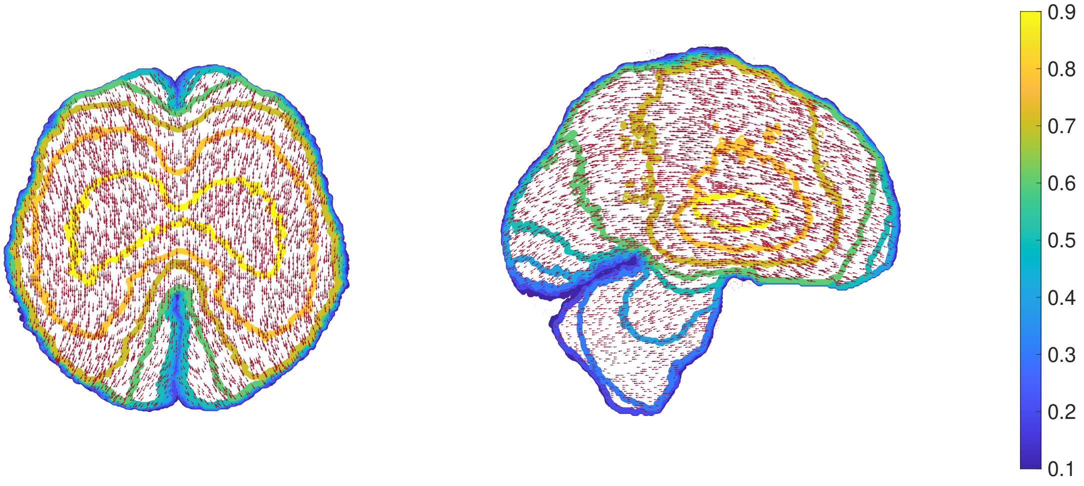

Figure 2. Axial (left) and sagittal (right) views of the synthetic displacement field used for the evaluation of the accuracy of the measurements of PBS. Length of vectors have been scaled for visualisation purposes: their magnitude is represented by the underlying contour plots (in [mm]).

Method	Parameter	Values
SyN	base knot spacing<br>neighbourhood<br>step update	16, 19, 23, 26, 29, 33<br>1,2,3,4,5,6<br>0.05,0.1,0.17,0.25,0.3
elastix	final grid spacing<br>spatial samples<br>region size	2 4 6 8<br>$1e^3$ $1.5e^3$ $2e^3$ $2.5e^3$<br>30 40 50 60 70
DRAMMS	samples<br>regularisation<br>final knot spacing	3, 5, 7<br>0.1, 0.15, 0.2, 0.25<br>3, 5, 7, 9

Table 3. Search grids for the optimisation of each registration methods.

Method	Parameter	Correlation coefficient
SyN	base knot spacing<br>neighbourhood<br>step update	-0.1789<br>0.0731<br>0.0151
elastix	final grid spacing<br>spatial samples<br>region size	0.0919<br>-0.1293<br>0
DRAMMS	samples<br>regularisation<br>final knot spacing	0.0022<br>-0.0080<br>-0.0556

Table 4. Sensitivity analysis showing, for each registration method, the influence of parameters on the error.

Table 5 shows the best parameter set for each method, with the corresponding RMSE. Fig. 9 shows the boxplot of the error at different ROI corresponding to the best parameter set for each method. The distribution of the error for one of the subjects is reported on an axial slice in Fig. 10 for the three methods. SyN showed the best performance and was therefore used throughout the study. Evaluating accuracy on synthetic data represents a best case-scenario[21], as synthetic warp field, interpolation and noise pattern cannot reproduce the realistic conditions fully. However, given the lack of ground truth to test the accuracy on and the lack of expertise in identifying / placing fiducial landmarks, any further attempt in assessing the accuracy of the method were considered out of scope.

References

1. Monea, A. G. *et al.* Assessment of relative brain-skull motion in quasistatic circumstances by magnetic resonance imaging. *J. Neurotrauma* **29**, 2305–2317, DOI: 10.1089/neu.2011.2271 (2012).
2. Schnaudigel, S. *et al.* Positional brain deformation visualized with magnetic resonance morphometry. *Neurosurgery* **66**, 376–384, DOI: 10.1227/01.NEU.0000363704.74450.B4 (2010).
3. Jovicich, J. *et al.* Reliability in multi-site structural MRI studies: Effects of gradient non-linearity correction on phantom and human data. *Neuroimage* **30**, 436–443, DOI: 10.1016/j.neuroimage.2005.09.046 (2006).
4. Avants, B. B., Epstein, C. L., Grossman, M. & Gee, J. C. Symmetric diffeomorphic image registration with cross-correlation: Evaluating automated labeling of elderly and neurodegenerative brain. *Med. Image Anal.* **12**, 26–41, DOI: 10.1016/j.media.2007.06.004 (2008).
5. Watanabe, Y., Lee, C. K. & Gerbi, B. J. Geometrical accuracy of a 3-tesla magnetic resonance imaging unit in Gamma Knife surgery. *J. Neurosurg.* **105**, 190–193, DOI: 10.3171/sup.2006.105.7.190 (2006).
6. Karger, C. P., Höss, A., Bendl, R., Canda, V. & Schad, L. Accuracy of device-specific 2D and 3D image distortion correction algorithms for magnetic resonance imaging of the head provided by a manufacturer. *Phys. Med. Biol.* **51**, N253–61, DOI: 10.1088/0031-9155/51/12/N04 (2006).

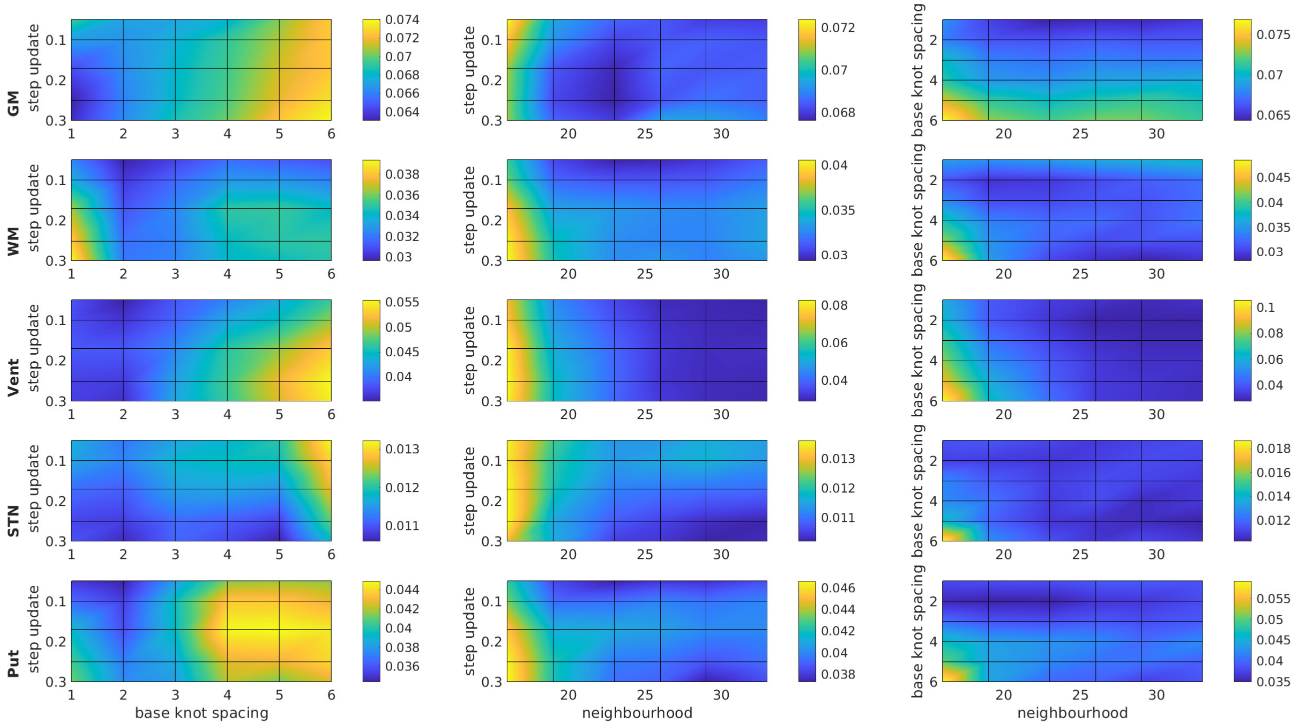

Figure 3. SyN: surface plots showing the distribution of the error over each pair of parameters at the following ROI: gray matter (GM), white matter (WM), ventricles (Vent) and putament (Put). Values are in [mm].

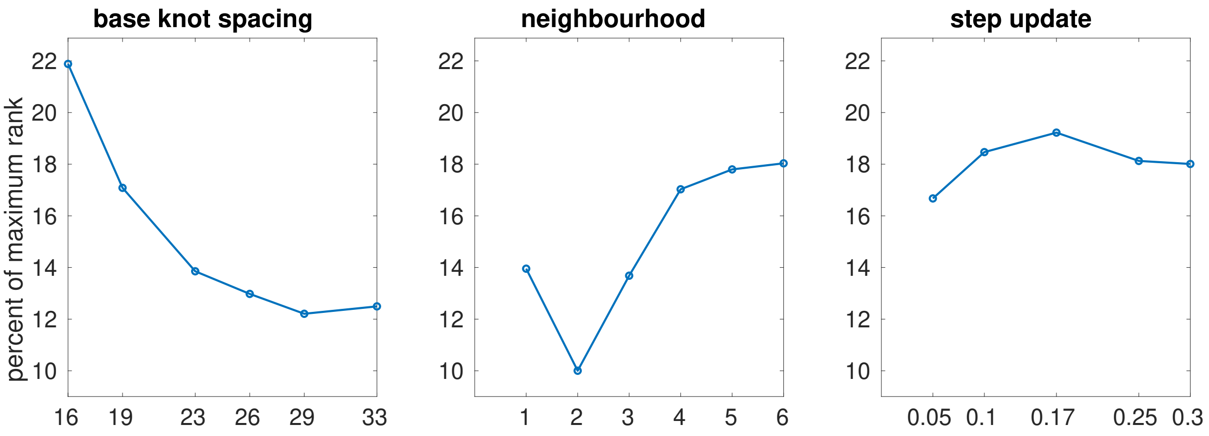

Figure 4. SyN: ranking values for each parameter. Ranks are reported as percentage of the maximum (i.e. worst) rank.

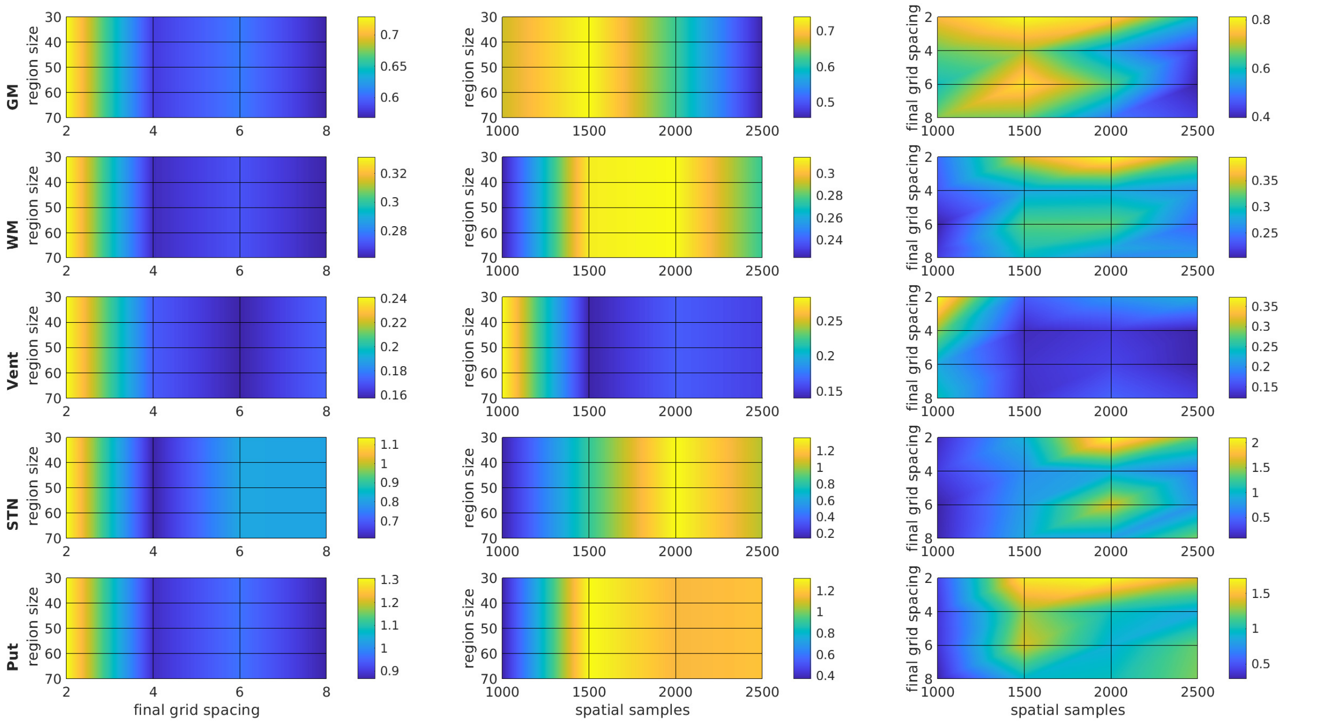

Figure 5. *elastix*: surface plots showing the distribution of the error over each pair of parameters at the following ROI: GM, WM, Vent, STN, Put. Values are in [mm].

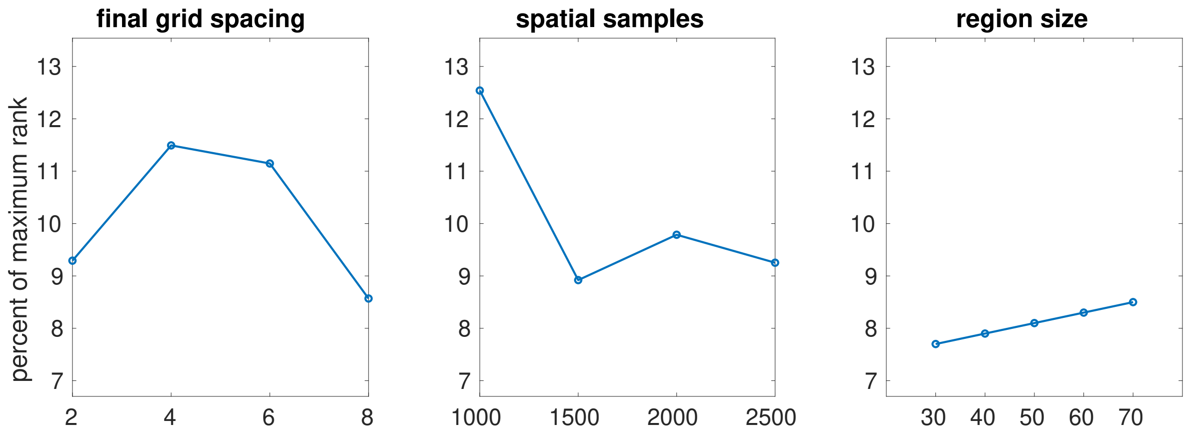

Figure 6. *elastix*: ranking values for each parameter. Ranks are reported as percentage of the maximum (i.e. worst) rank.

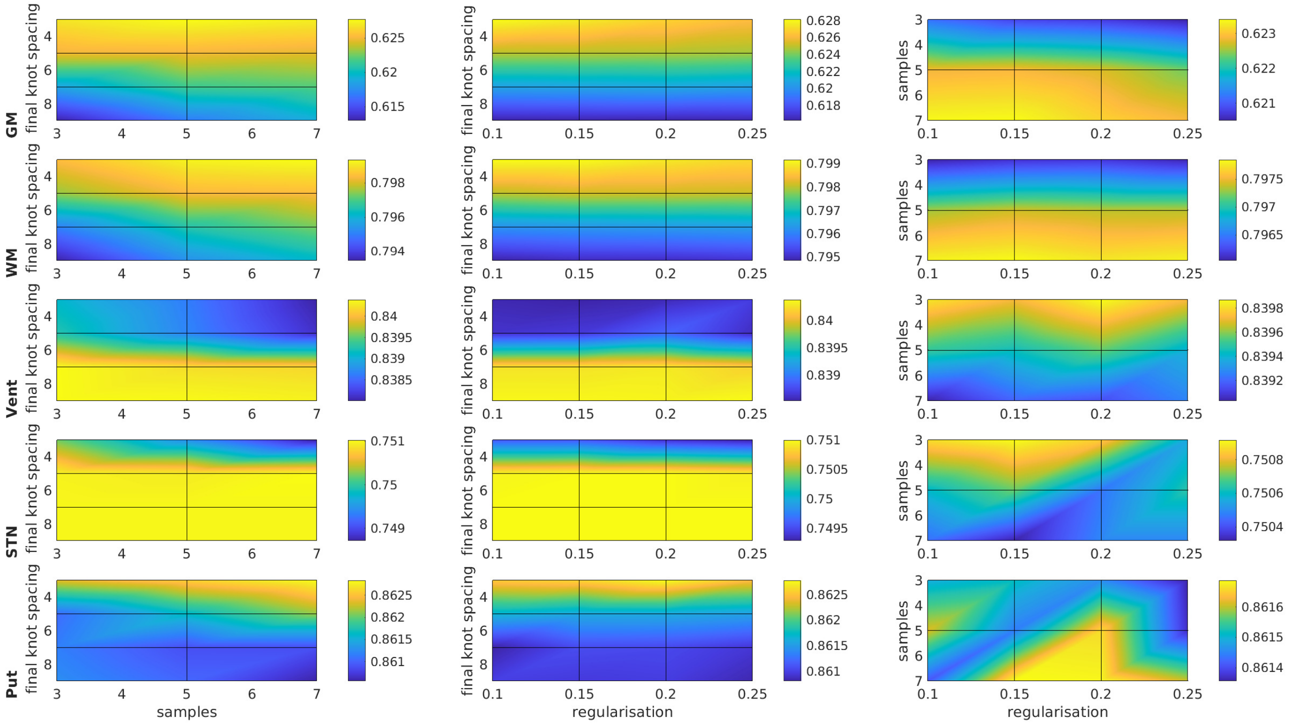

Figure 7. DRAMMS: surface plots showing the distribution of the error over each pair of parameters at the following ROI: GM, WM, Vent, STN, Put. Values are in [mm].

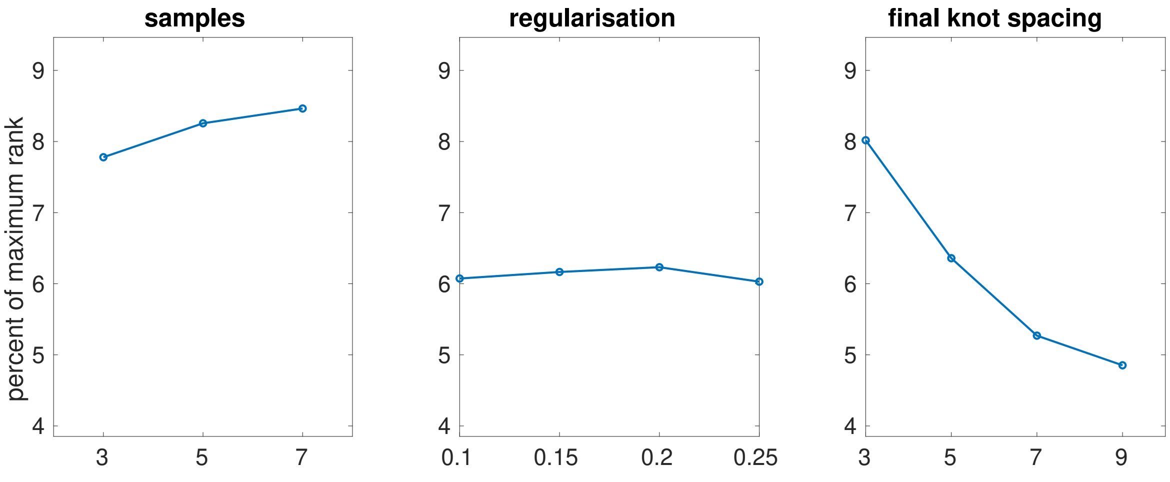

Figure 8. DRAMMS: ranking values for each parameter. Ranks are reported as percentage of the maximum (i.e. worst) rank.

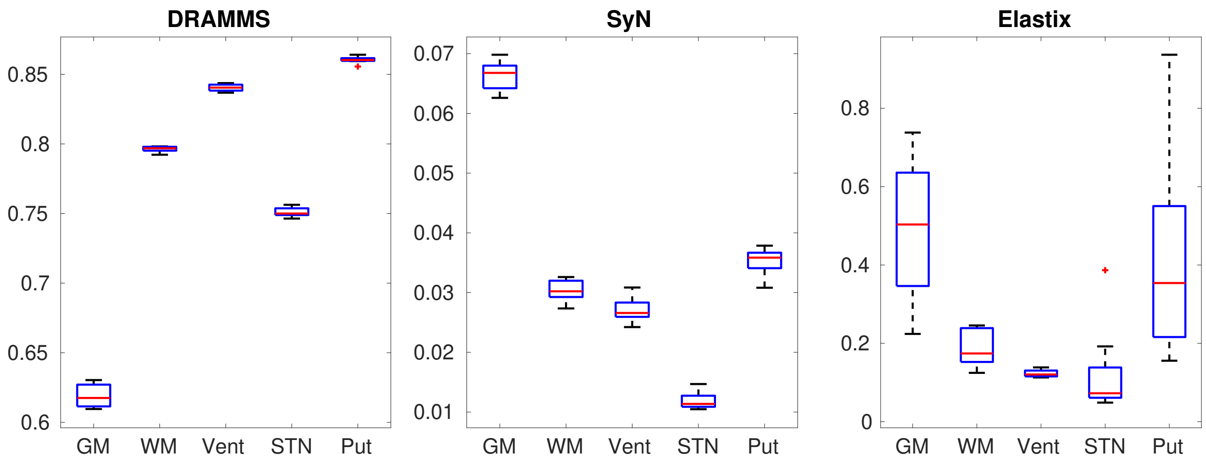

Figure 9. Boxplot of the mean squared error of each optimised registration algorithms averaged among the following ROI: (GM, WM, Vent, STN, Put).

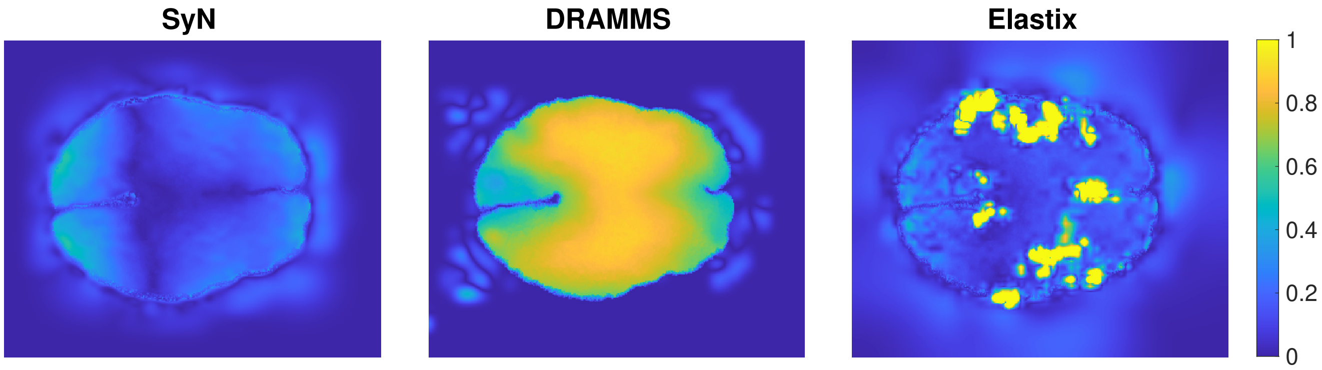

Figure 10. Distribution of the mean squared error corresponding to the best parameter set over the brain area for one subject. Colour bar represents values in [mm].

Method	Name	Value	mean ± std (5th percentile, 95th percentile) error
SyN	base knot spacing<br>neighbourhood<br>step update	29<br>20<br>0.05	0.0342 ± 0.0182 (0.0109, 0.0680) mm
elastix	final grid spacing<br>spatial samples<br>region size	4<br>1500<br>30	0.2683 ± 0.2140 (0.0611, 0.7305) mm
DRAMMS	samples<br>regularisation<br>final knot spacing	7<br>0.25<br>9	0.7734 ± 0.0871 (0.6115, 0.8616) mm

Table 5. Best parameter set for each registration methods with the corresponding error averaged over the brain area.

7. Tavares, W. M. *et al.* An image correction protocol to reduce distortion for 3-T stereotactic MRI. *Neurosurgery* **74**, 121–126, DOI: 10.1227/NEU.0000000000000178 (2014).

8. Neumann, J. O., Giese, H., Biller, A., Nagel, A. M. & Kiening, K. Spatial Distortion in MRI-Guided Stereotactic Procedures: Evaluation in 1.5-, 3- and 7-Tesla MRI Scanners. *Stereotact. Funct. Neurosurg.* **93**, 380–386, DOI: 10.1159/000441233 (2015).

9. Jenkinson, M., Bannister, P., Brady, M. & Smith, S. Improved Optimization for the Robust and Accurate Linear Registration and Motion Correction of Brain Images. *Neuroimage* **17**, 825–841, DOI: 10.1006/nimg.2002.1132 (2002).

10. Klein, S., Staring, M., Murphy, K., Viergever, M. A. & Pluim, J. P. Elastix: A toolbox for intensity-based medical image registration. *IEEE Trans. Med. Imaging* **29**, 196–205, DOI: 10.1109/TMI.2009.2035616 (2010).

11. Dice, L. R. Measures of the Amount of Ecologic Association Between Species. *Ecology* **26**, 297–302, DOI: 10.2307/1932409 (1945).

12. Klein, A. *et al.* Evaluation of 14 nonlinear deformation algorithms applied to human brain MRI registration. *Neuroimage* **46**, 786–802, DOI: 10.1016/j.neuroimage.2008.12.037 (2009).

13. Ou, Y., Akbari, H., Bilello, M., Da, X. & Davatzikos, C. Comparative evaluation of registration algorithms in different brain databases with varying difficulty: Results and insights. *IEEE Trans. Med. Imaging* **33**, 2039–2065, DOI: 10.1109/TMI.2014.2330355 (2014).

14. Murphy, K. *et al.* Evaluation of registration methods on thoracic CT: The EMPIRE10 challenge. *IEEE Trans. Med. Imaging* **30**, 1901–1920, DOI: 10.1109/TMI.2011.2158349 (2011).

15. Avants, B., Tustison, N. & Song, G. Advanced Normalization Tools (ANTS). *Insight J.* 1–35 (2009).

16. Ou, Y., Sotiras, A., Paragios, N. & Davatzikos, C. DRAMMS: Deformable registration via attribute matching and mutual-saliency weighting. *Med. Image Anal.* **15**, 622–639, DOI: 10.1016/j.media.2010.07.002 (2011).

17. Sotiras, A., Davatzikos, C. & Paragios, N. Deformable medical image registration: A survey. *IEEE Trans. Med. Imaging* **32**, 1153–1190, DOI: 10.1109/TMI.2013.2265603 (2013).

18. Holden, M. A review of geometric transformations for nonrigid body registration. *IEEE Trans. Med. Imaging* **27**, 111–128, DOI: 10.1109/TMI.2007.904691 (2008).

19. Bennion, N. *Computational modelling of brain shift in stereotactic neurosurgery.* Ph.D. thesis, Cardiff University (2020).

20. Staring, M. *et al.* Towards local progression estimation of pulmonary emphysema using CT. *Med. Phys.* **41**, 021905, DOI: 10.1118/1.4851535 (2014).

21. Pluim, J. P., Muenzing, S. E., Eppenhof, K. A. & Murphy, K. The truth is hard to make: Validation of medical image registration. In *2016 23rd Int. Conf. Pattern Recognit.*, vol. 0, 2294–2300, DOI: 10.1109/ICPR.2016.7899978 (2016).
